# Author Correction: Significant underestimation of radiative forcing by aerosol–cloud interactions derived from satellite-based methods

**DOI:** 10.1038/s41467-021-24518-6

**Published:** 2021-07-06

**Authors:** Hailing Jia, Xiaoyan Ma, Fangqun Yu, Johannes Quaas

**Affiliations:** 1 Collaborative Innovation Center on Forecast and Evaluation of Meteorological Disasters, and Key Laboratory for Aerosol-Cloud-Precipitation of China Meteorological Administration, School of Atmospheric Physics, Nanjing University of Information Science & Technology, Nanjing, China; 2 Atmospheric Sciences Research Center, University at Albany, Albany, NY USA; 3 Institute for Meteorology, Universität Leipzig, Leipzig, Germany

**Keywords:** Climate sciences, Atmospheric science, Climate change

Correction to: *Nature Communications* 10.1038/s41467-021-23888-1, published online 15 June 2021.

The original version of this Article contained an error in the acknowledgments.

‘This research has been supported by the National Key R&D Program of China (grant nos. 2019YFA0606802 and 2016YFA0600404)’ should have read ‘This research has been supported by the National Key R&D Program of China (grant no. 2019YFA0606802)’.

This has now been corrected in both the PDF and HTML versions of the Article.

