# Supplementary material for: Significant underestimation of radiative forcing by aerosol–cloud interactions derived from satellite-based methods
Source: Nat Commun. 2021 Jun 15;12:3649. doi: 10.1038/s41467-021-23888-1 (PMC8206093; doi:10.1038/s41467-021-23888-1)
Supplement: Supplementary file 1 — Supplementary Information [file 41467_2021_23888_MOESM1_ESM.pdf]

# Supplementary information to "Significant underestimation of radiative forcing by aerosol–cloud interactions derived from satellite-based methods"

Hailing Jia[1,2,3], Xiaoyan Ma[1*], Fangqun Yu [2*] & Johannes Quaas[3]

[1]Collaborative Innovation Center on Forecast and Evaluation of Meteorological Disasters, and Key Laboratory for Aerosol-Cloud-Precipitation of China Meteorological Administration, School of Atmospheric Physics, Nanjing University of Information Science & Technology, Nanjing, China

[2]Atmospheric Sciences Research Center, University at Albany, Albany, NY, USA

[3] Institute for Meteorology, Universität Leipzig, Leipzig, Germany

**Supplementary Table 1.** Modeled and satellite-based $RF_{aci}$ (W $m^{-2}$) for different regions, respectively.

| Region | Modeled $RF_{aci}$ (W $m^{-2}$) | Satellite-based $RF_{aci}$ (W $m^{-2}$) | | | |
|---|---|---|---|---|---|
| | | Aero_Cld_Modis | Aero_Cld | Cld | All_Cld |
| Global | -0.79 | -0.36 | -0.38 | -0.75 | -0.59 |
| NH | -1 | -0.57 | -0.62 | -1.21 | -0.98 |
| SH | -0.57 | -0.15 | -0.14 | -0.28 | -0.19 |
| Land | -1.23 | -0.24 | -0.33 | -1.22 | -0.77 |
| Ocean | -0.64 | -0.4 | -0.4 | -0.59 | -0.53 |
| NPO | -0.94 | -1.54 | -1.67 | -2.22 | -2.17 |
| NAM | -1.71 | -0.33 | -0.49 | -2.12 | -1.4 |
| NAO | -1.45 | -0.59 | -0.59 | -1.38 | -1.05 |
| EUR | -2.54 | -0.7 | -0.9 | -1.86 | -1.31 |
| ASI | -2.22 | -0.4 | -0.58 | -1.74 | -1.3 |
| TPO | -0.39 | -0.22 | -0.17 | -0.21 | -0.22 |
| TAO | -0.51 | -0.32 | -0.31 | -0.45 | -0.42 |
| AFR | -0.35 | -0.12 | -0.1 | -0.61 | -0.26 |
| TIO | -0.15 | -0.14 | -0.09 | -0.28 | -0.14 |
| SPO | -0.69 | -0.11 | -0.1 | -0.09 | -0.07 |
| SAM | -0.6 | -0.01 | -0.07 | -0.94 | -0.4 |
| SAO | -0.64 | -0.17 | -0.19 | -0.2 | -0.2 |
| SIO | -0.57 | -0.2 | -0.18 | -0.19 | -0.19 |
| OCE | -0.54 | -0.04 | -0.12 | -0.22 | -0.16 |

**Supplementary Table 2.** The list of the parameters, sources, and their corresponding temporal-spatial resolutions applied in present study.

| Source | Time period | Temporal-spatial resolution | Parameters |
|---|---|---|---|
| CERES-SSF Edition 4A[1] | Jan 2002–Dec 2018 | Daily, approximately 20×20 $km^2$ | Surface type<br>Surface-type percent coverage<br>Solar zenith angle<br>Shortwave TOA flux–upward<br>Cloud fraction ($f$) [a]<br>Cloud phase<br>Cloud optical depth @3.7 μm ($\tau_c$)<br>Cloud effective radius @3.7 μm ($r_e$)<br>Cloud liquid water path ($L$) |
| MODIS C61[2] | Jan 2002–Dec 2018 | Daily, 1° × 1° | AOD @550 nm [b] |
| POLDER-3[3] | Mar 2005–Oct 2013 | Daily, 1° × 1° | AOD & $AOD_f$ @565 nm |
| MERRA-2[4] | Jan 2002–Dec 2018 | Hourly, 0.5°×0.625° | AOD @550 nm<br>Sulfate AOD@550 nm<br>Black carbon AOD@550 nm<br>Organic carbon AOD@550 nm<br>Sea salt AOD@550 nm<br>Angström exponent[c] (470-870 nm)<br>Sulfate column mass density (SO4) |
| MACv2[5] | | Monthly, 1° × 1° | Anthropogenic fractions of $AOD_f$ from AeroCom phase1[6] and phase2[7] models |

[a] $f$ is derived by clear area percent coverage.

[b] MODIS AOD is from the Dark Target and Deep Blue merged aerosol product.

[c] Angström exponent is used to calculate aerosol index (AI = AOD×Angström exponent)

**Supplementary Table 3**. Global (60°S to 60°N) annual mean MERRA-2 daily-based and POLDER-3 monthly-based $RF_{aci}$ (in W $m^{-2}$) estimated with different anthropogenic fractions, respectively. The corresponding global annual averages of anthropogenic fractions of $AOD_f$ are also shown. The associated spatial distributions are given in Supplementary Figure 9.

| Source | Anthropogenic fractions of $AOD_f$ | MERRA-2 daily-based $RF_{aci}$ | POLDER-3 monthly-based $RF_{aci}$ |
|---|---|---|---|
| AeroCom 1 | 0.49 | −1.68 | −1.67 |
| AeroCom 2 | 0.37 | −1.07 | −1.08 |
| GC-APM | 0.40 | −1.41 | −1.42 |
| GC-APM-SS30 | 0.32 | −1.09 | −1.02 |

*Anthropogenic fractions of $AOD_f$ are acquired from the simulations of (1) AeroCom phase1 models[6] with the preindustrial year 1750 as reference (AeroCom 1), (2) AeroCom phase2 models[7] with the preindustrial year 1850 as reference (AeroCom 2), (3) GEOS-Chem-APM[8] with $AOD_f$ defined as the sum of AODs of sulfate, black carbon and organic aerosol (GC-APM), and (4) GEOS-Chem-APM with $AOD_f$ defined as the sum of AODs sulfate, black carbon and organic aerosol and 30 % sea salt aerosol (GC-APM-SS30), respectively.

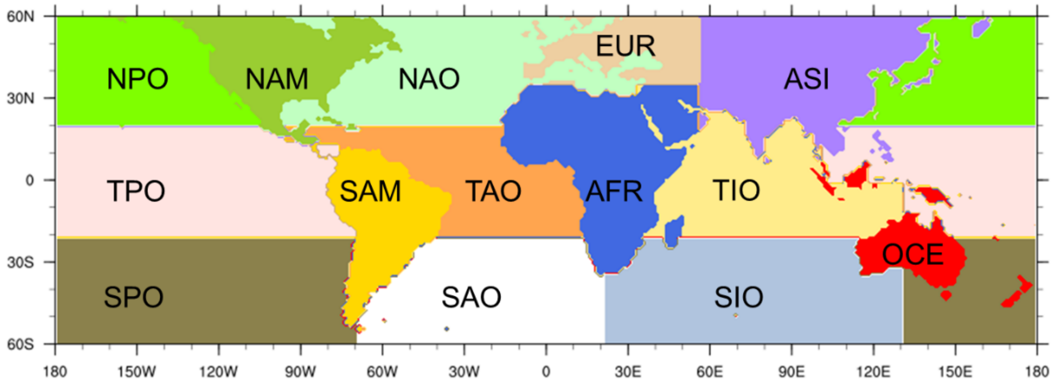


**Supplementary Figure 1. The fourteen different regions used in this study.** NPO: North Pacific Ocean; NAM: North America; NAO: North Atlantic Ocean; EUR: Europe; ASI: Asia; TPO: Tropical Pacific Ocean; TAO: Tropical Atlantic Ocean; AFR: Africa; TIO: Tropical Indian Ocean; SPO: South Pacific Ocean; SAM: South America; SAO: South Atlantic Ocean; SIO: South Indian Ocean; and OCE: Australia.

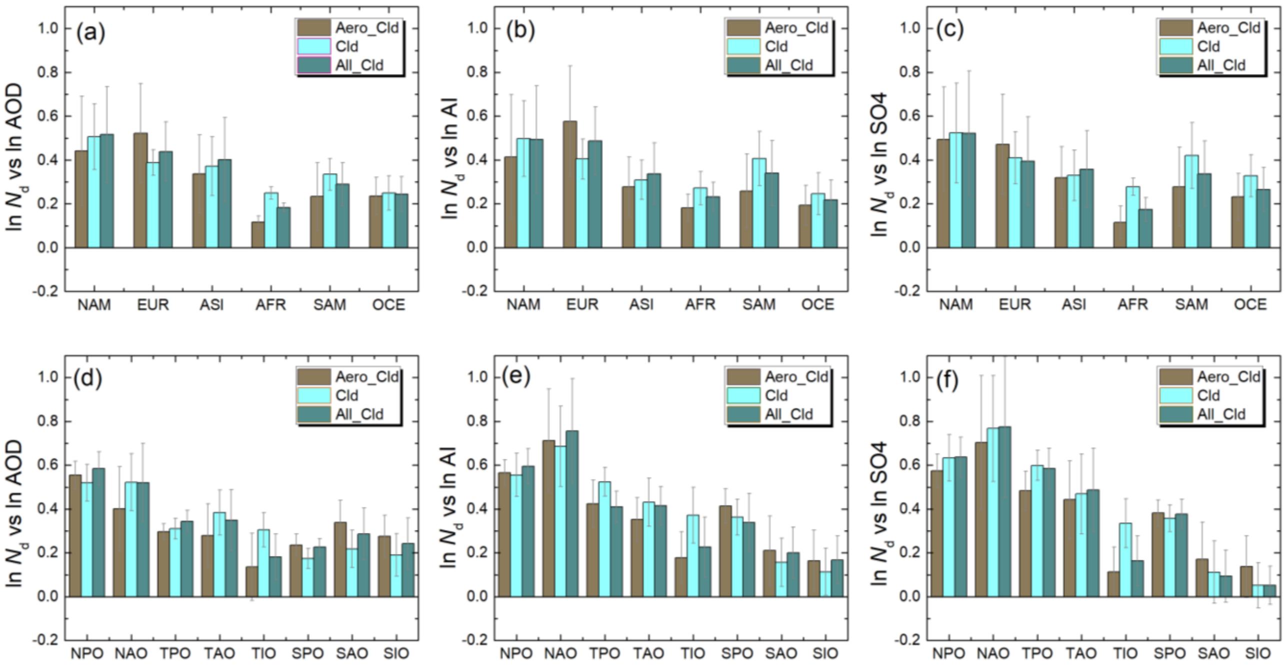


**Supplementary Figure 2. Annual averaged slopes of the linear regressions between the logarithm of cloud droplet number concentration ($N_d$) and those of cloud condensation nuclei (CCN) proxies, including aerosol optical depth (AOD), aerosol index (AI), and sulfate column mass concentration (SO4).** Shown are the slopes of **a, d** ln $N_d$ versus ln AOD, **b, e** ln $N_d$ versus ln AI, and **c, f** ln $N_d$ versus ln SO4 for the regions over land (top) and ocean (bottom). Annual averaged slope here is the average of the monthly slopes. The standard deviation of the inter-monthly variability of the regression slopes is shown as an error bar.

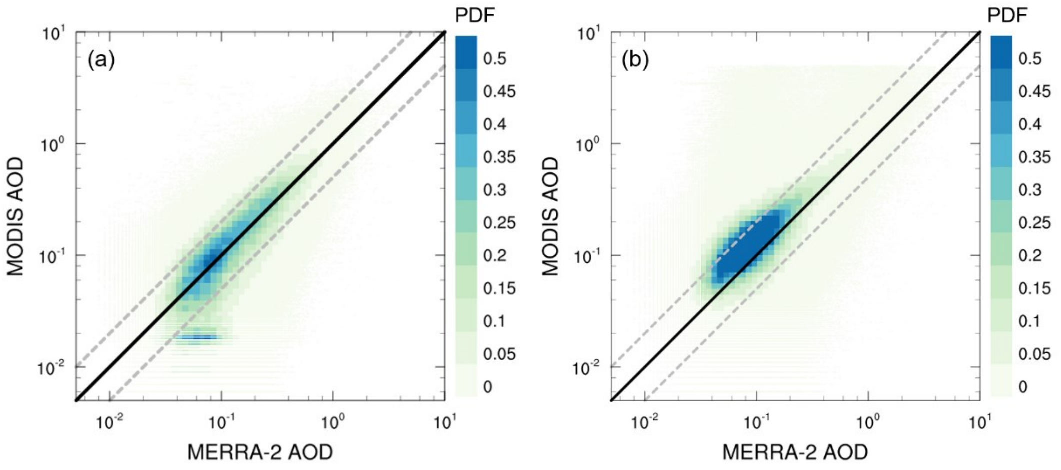


**Supplementary Figure 3. Comparison of MERRA-2 and MODIS AOD for the year of 2010**. **a** and **b** are for clear-sky and cloudy conditions, respectively. Color is coded by joint probability distribution function (PDF). The black line indicates the 1:1 line, and the gray dashed ones represent the lines of y = 2x and y= 0.5x, respectively.

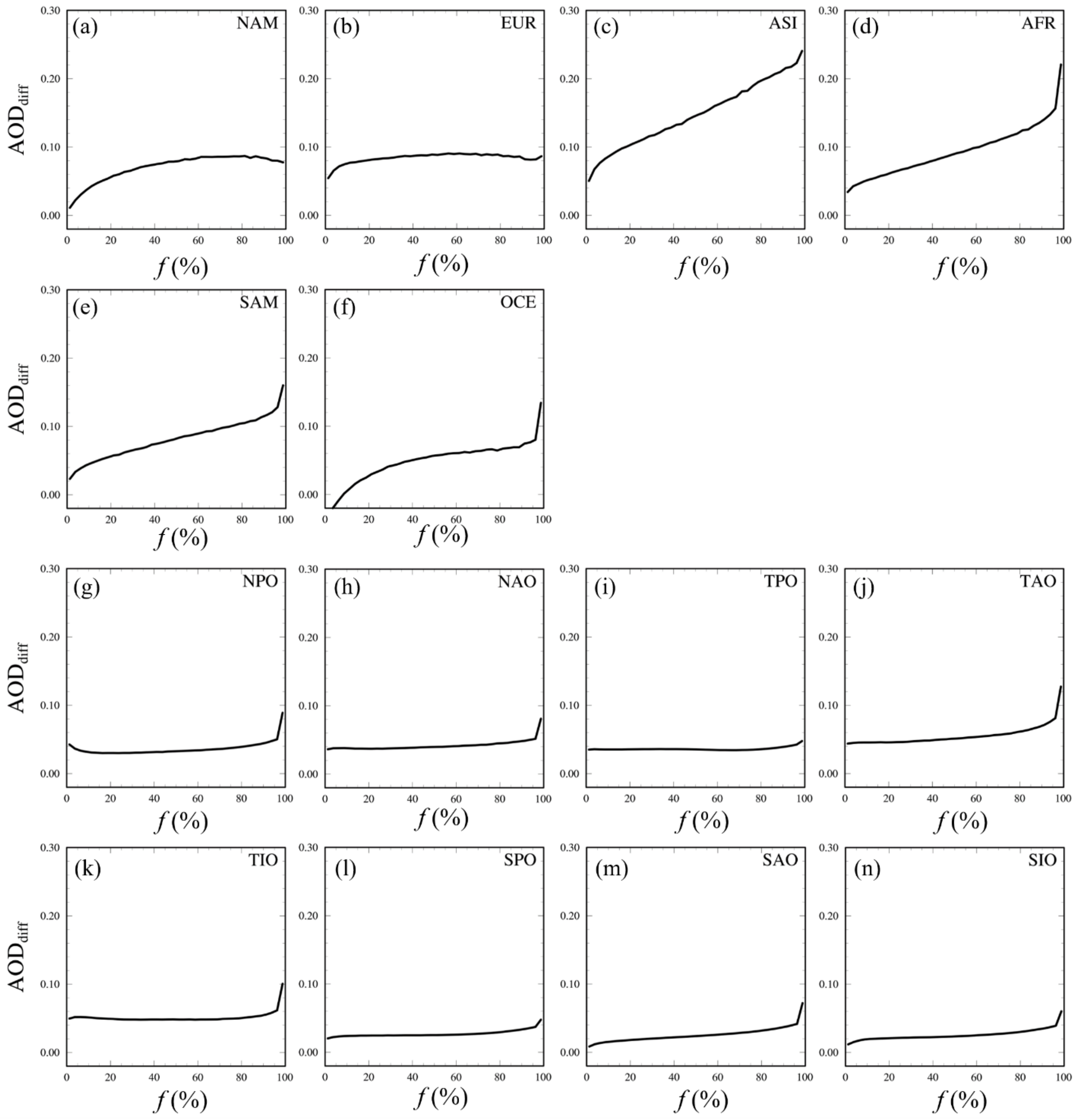


**Supplementary Figure 4. The difference between MODIS AOD and MERRA-2 AOD as function of cloud fraction (*f*). a-n** are for the fourteen regions as defined in Supplementary Figure 1.

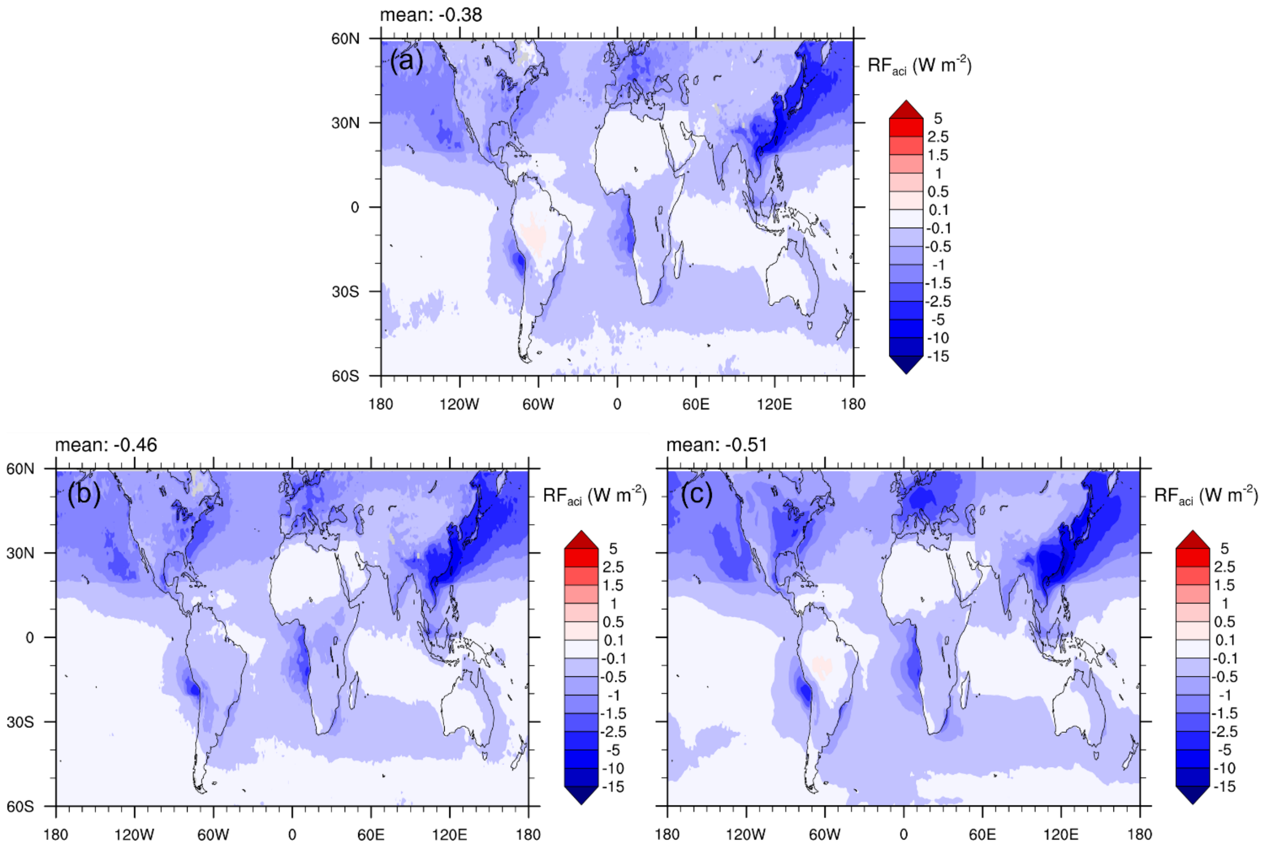


**Supplementary Figure 5. Annual mean first indirect forcing ($RF_{aci}$) for three different scenarios. a** The scenario that MODIS aerosol and cloud retrievals are simultaneously successful (Aero_Cld), **b** same cloud samples as Aero_Cld but using the regression coefficients for the scenario including all ambient clouds (Aero_Cld_R), which is designed to evaluate the effect of changed regression coefficients, and **c** using same regression coefficients as Aero_Cld but including all ambient clouds when computing $RF_{aci}$ (Aero_Cld_C), which is designed to quantify the impact of changed cloud fraction. Aerosol optical depth used in all cases are from MERRA-2 reanalysis data.

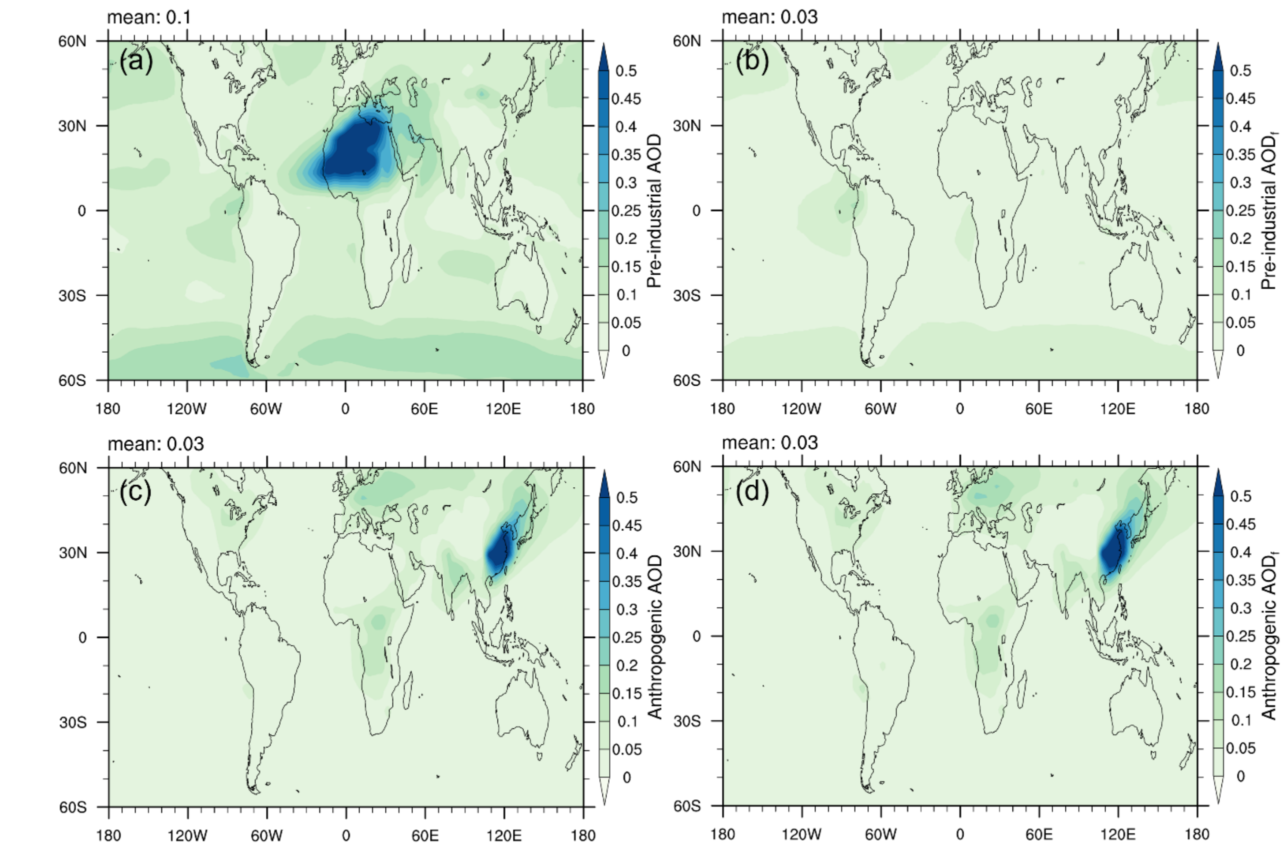


**Supplementary Figure 6. Annual (2010) averaged pre-industrial and anthropogenic aerosol optical depth (AOD) and fine mode AOD ($AOD_f$).** Shown are pre-industrial **a** AOD and **b** $AOD_f$, and **c** anthropogenic AOD and **d** $AOD_f$ from the GEOS-Chem-APM simulations.

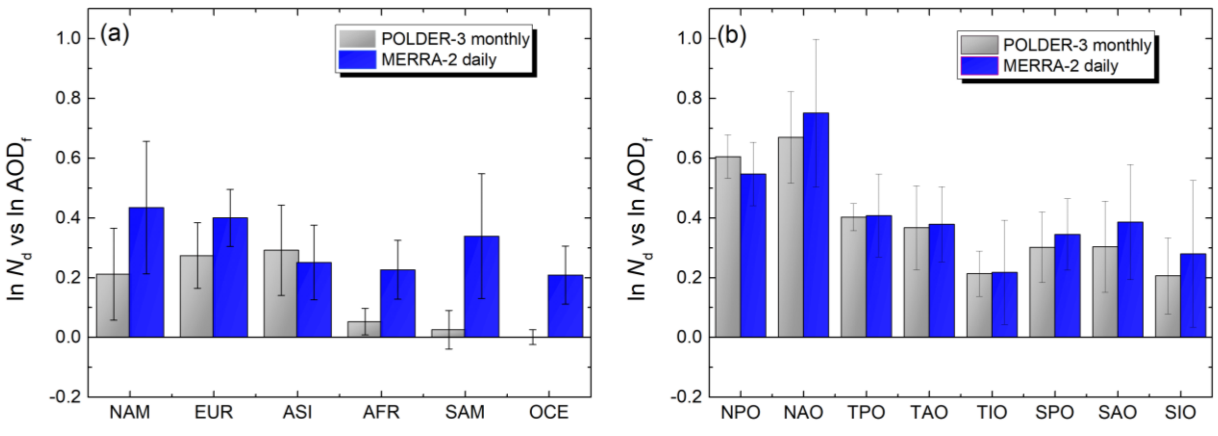


**Supplementary Figure 7. Annually averaged slopes of the linear regressions between the logarithm of cloud droplet number concentration ($N_d$) and that of fine mode aerosol optical depth ($AOD_f$) for the fourteen regions.** Grey and blue bars represent the slopes based on POLDER-3 monthly statistics and MERRA-2 daily (for the scenario including all ambient clouds, i.e., All_Cld) statistics over **a** continental and **b** oceanic regions, respectively. Both cases can sidestep the influence of sampling biases. The annually averaged slope here is the average of the monthly slopes. The standard deviation of the inter-monthly variability of the regression slopes is shown as an error bar.

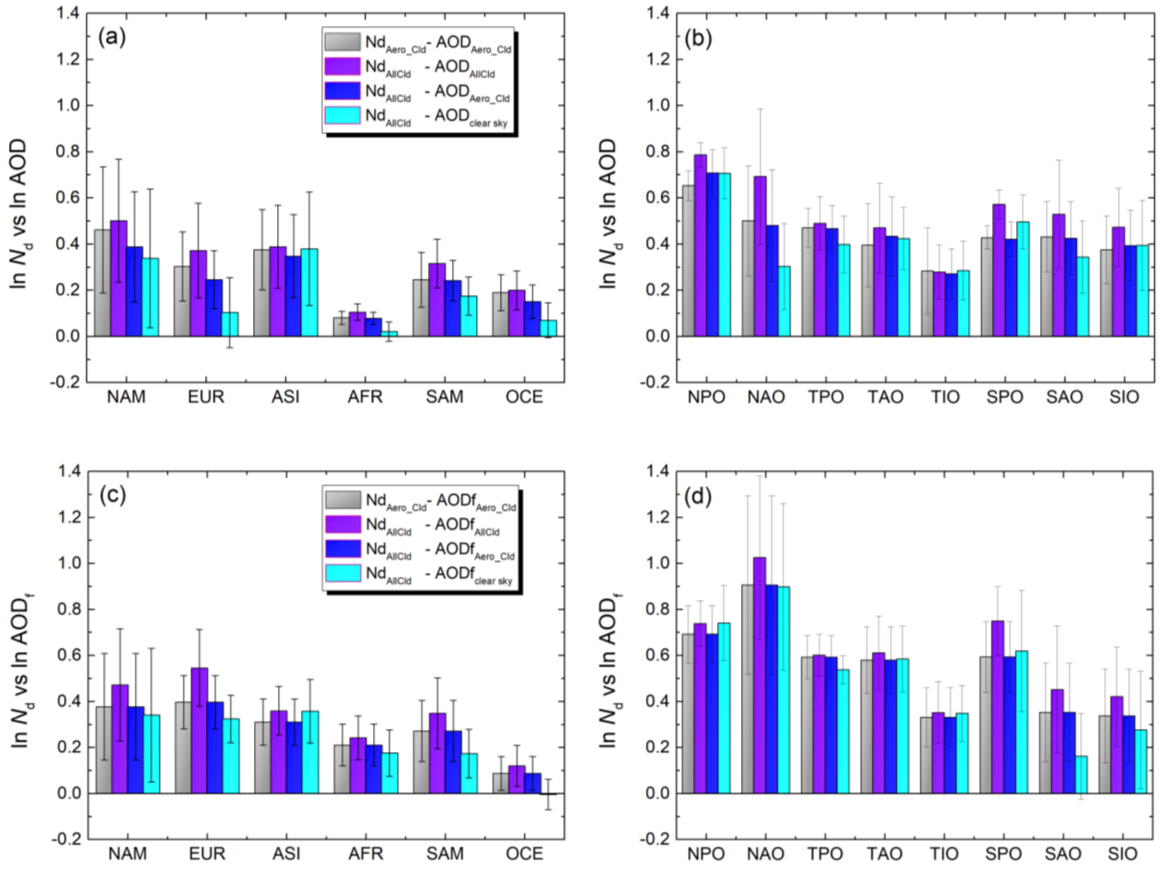


**Supplementary Figure 8. Annually averaged slopes of the linear regressions between the logarithm of cloud droplet number concentration ($N_d$) and those of aerosol optical depth (AOD) and fine mode AOD ($AOD_f$) based on monthly aerosol-cloud associations.** Shown are **a, b** ln $N_d$ versus ln AOD and **c, d** ln $N_d$ versus $AOD_f$ for the fourteen regions. Here, AOD ($AOD_f$) is from MERRA-2 re-analysis data. Monthly $N_d$ and AOD ($AOD_f$) statistics are subsampled from daily data according to four strategies as indicated by different subscripts (AllCld, AeroCld, clear sky). $N_{dAllCld}$ ($AOD_{AllCld}$ /$AODf_{AllCld}$) represents the monthly $N_d$ (AOD / $AOD_f$) calculated from the data including all clouds. $N_{dAeroCld}$ ($AOD_{AeroCld}$ /$AODf_{AeroCld}$) represents the monthly $N_d$ (AOD / $AOD_f$) calculated from the subset of data where aerosol and cloud retrievals are available simultaneously. Here the 'clear sky' case includes not only the AOD ($AOD_f$) with $f$ = 0 but also the AOD ($AOD_f$) without successful $N_d$ retrieval over entire 1°x1° grid box, which is used to represent the monthly AOD ($AOD_f$) that is irrelevant to monthly $N_{dAllCld}$. The annually averaged slope is the average of the monthly slopes. The standard deviation of the inter-monthly variability of the regression slopes is shown as an error bar.

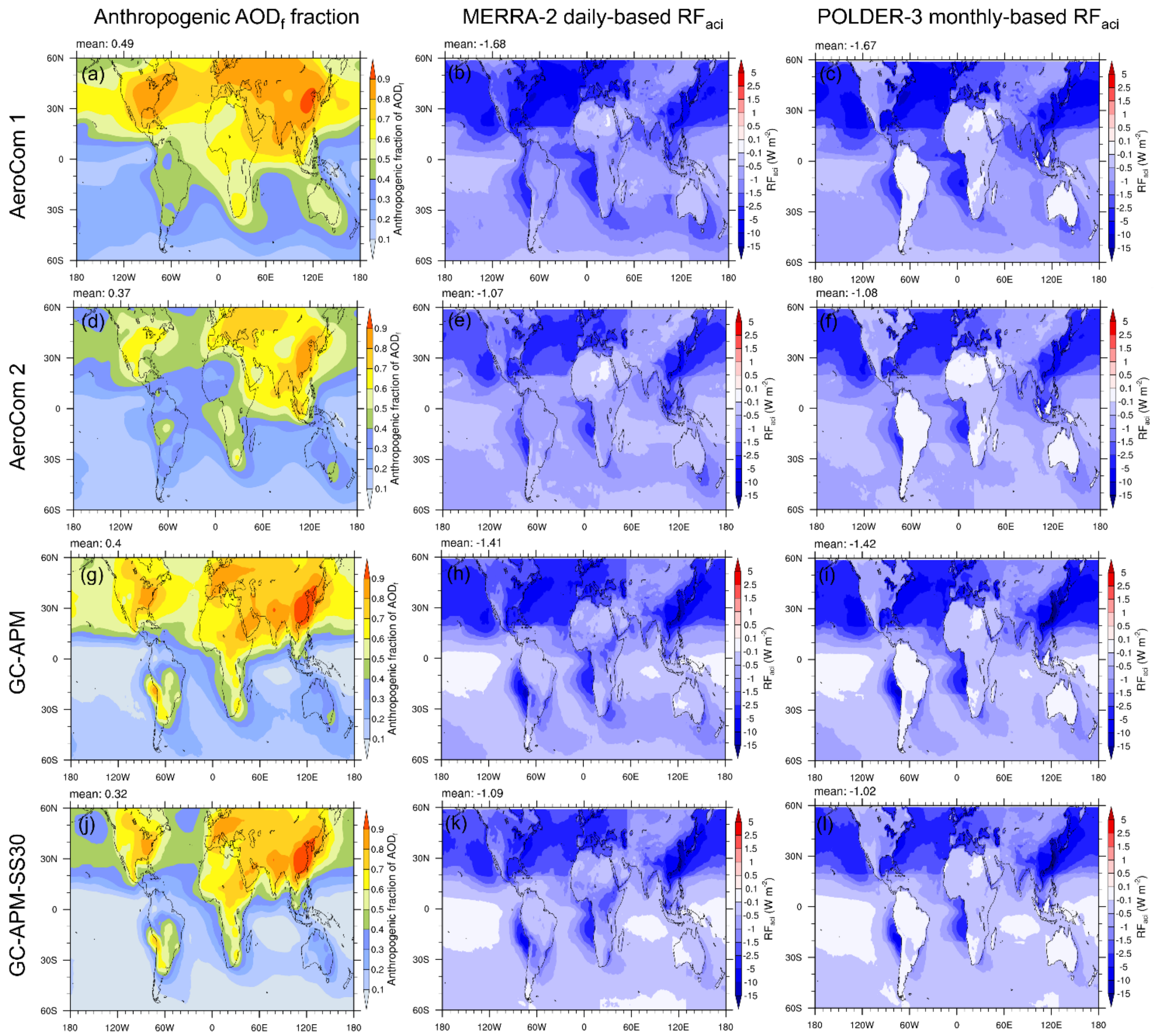


**Supplementary Figure 9. Annual mean anthropogenic fractions and their associated first indirect forcing ($RF_{aci}$) based on daily and monthly aerosol-cloud associations, respectively.** Shown on the left column are anthropogenic fractions of fine mode aerosol optical depth ($AOD_f$) acquired from the simulations of **a** AeroCom phase1 models with the preindustrial year 1750 as reference (AeroCom 1), **d** AeroCom phase2 models with the preindustrial year 1850 as reference (AeroCom 2), **g** GEOS-Chem-APM with $AOD_f$ defined as the sum of AODs of sulfate, black carbon and organic aerosol (GC-APM), and **j** GEOS-Chem-APM with $AOD_f$ defined as the sum of AODs sulfate, black carbon and organic aerosol and 30 % sea salt aerosol (GC-APM-SS30), respectively. **b, e, h, k** MERRA-2 daily-based and **c, f, i, l** POLDER-3 monthly-based $RF_{aci}$ estimated with above-mentioned four anthropogenic $AOD_f$ fractions are shown on the middle and right columns, respectively.

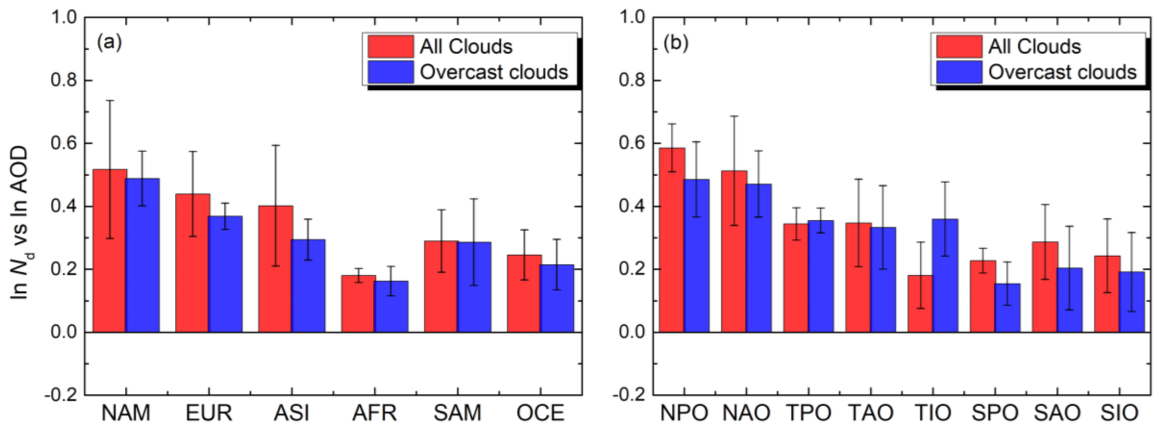


**Supplementary Figure 10. Annually averaged slopes of the linear regressions between the logarithm of cloud droplet number concentration ($N_d$) and that of MERR-2 aerosol optical depth (AOD) for the fourteen regions.** Red and blue bars represent the results based on all cloud samples and overcast clouds only, respectively. Here, the overcast pixel is defined as a pixel with 20×20 km$^2$ resolved cloud fraction larger than 80 %. The annually averaged slope here is the average of the monthly slopes. The standard deviation of the inter-monthly variability of the regression slopes is shown as an error bar.

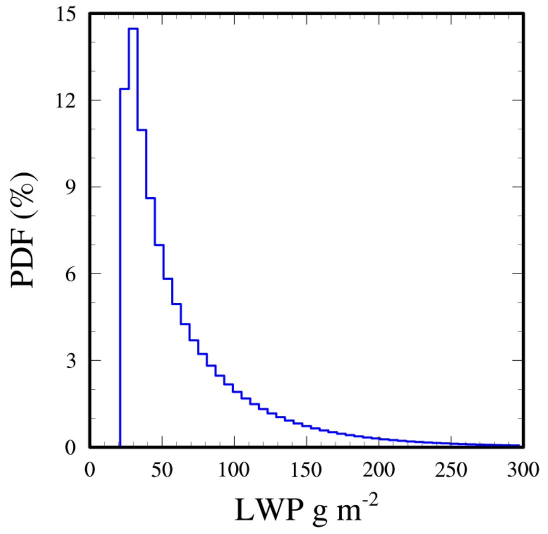


**Supplementary Figure 11. Global probability density function of liquid water path (LWP) over the period 2002 − 2018.**

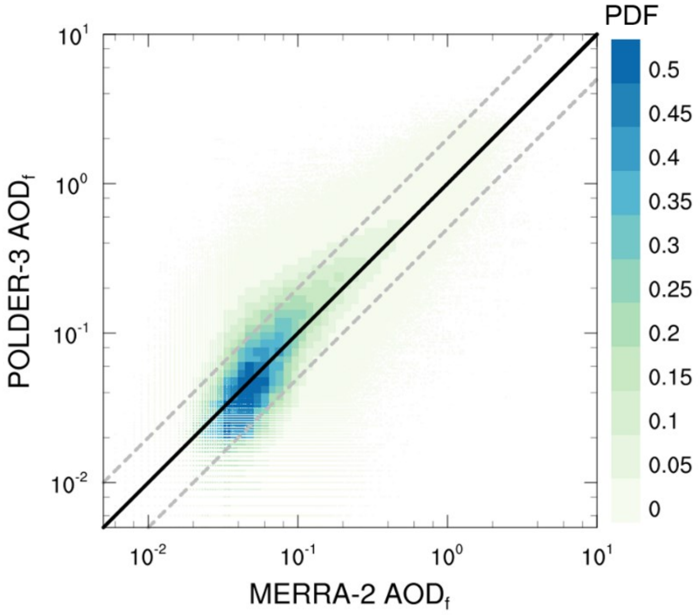


**Supplementary Figure 12. Comparison of MERRA-2 and POLDER-3 AOD$_f$ for the year of 2010.** Color is coded by the joint probability distribution function (PDF). The black line indicates the 1:1 line, and the gray dashed ones represent the lines of y = 2x and y= 0.5x, respectively.

## Supplementary References


1. Wielicki, B. A. et al. Clouds and the Earth's radiant energy system (CERES): An Earth observing system experiment. *B. Am. Meteorol. Soc.* **77**, 853–868 (1996).
2. Levy, R.C. et al. The Collection 6 MODIS aerosol products over land and ocean. *Atmos. Meas. Tech*. **6**, 2989–3034 (2013).
3. Dubovik, O. et al. GRASP: a versatile algorithm for characterizing the atmosphere. *SPIE Newsroom*, **25** (2014)
4. Randles, C. A. et al. The MERRA-2 aerosol reanalysis, 1980 onward. Part I: System description and data assimilation evaluation. *J. Climate.* **30**, 6823-6850 (2017).
5. Kinne, S. The MACv2 aerosol climatology. *Tellus B* **71**, 1-21 (2019).
6. Kinne, S. et al. An AeroCom initial assessment optical properties in aerosol component modules of global models. *Atmos. Chem. Phys.* **6**, 1815-1834 (2006).
7. Myhre, G. et al. Radiative forcing of the direct aerosol effect from AeroCom Phase II simulations. *Atmos. Chem. Phys.* **13**, 1853-1877 (2013).
8. Yu, F. & Luo, G. Simulation of particle size distribution with a global aerosol model: Contribution of nucleation to aerosol and CCN number concentrations. *Atmos. Chem. Phys.* **9**, 7691–7710 (2009).
